# Prevention and monitoring of metabolic syndrome in patients with severe mental ilness - presentation of our study protocol

**DOI:** 10.1192/j.eurpsy.2022.1950

**Published:** 2022-09-01

**Authors:** J. Lopes, T. Reis

**Affiliations:** Hospital Espirito Santo, Departamento De Psiquiatria E Saude Mental, Evora, Portugal

**Keywords:** prevention, metabolic syndrome, cardiovascular risk

## Abstract

**Introduction:**

Cardiovascular diseases are the leading cause of death in individuals with SMI. The sedentary lifestyle that usually guides these individuals associated with the use of some psychotropic drugs increases the risk of adverse events related to these pathologies

**Objectives:**

Presentation of the Study Protocol for the Implementation of a Psychoeducational Group directed to Prevention and Monitoring Metabolic Syndrome (MS) in patients with SMI

**Methods:**

It is intended to implement a psychoeducational program, which includes a 30-minute walk, focused on healthy lifestyle habits, for 16 weeks.

It is intended to include SMI individuals, from a convenience sample, who present any of these criteria: Excess weight; At risk or diagnosed with DM; Sedentary lifestyle; Smoker. Data regarding socio-demographic, clinical and motivational for and about physical activity will be collected from the intervention group. Patients who refuse to join the study will only receive information about lifestyle changes at the beginning and will continue with their usual care.

**Results:**

According to available literature, it is expected that the monitoring and control of these parameters will translate into a benefit in reducing cardiovascular risk factors and optimizing the treatment of MS, contributing to the empowerment of patients in managing their disease and increasing their quality and years of life.

**Conclusions:**

The impact of lifestyle changes proved to be effective and are sometimes lasting, with objective gains in quality of life of these patients. The main measure to face this issue, improve the well-being and physical health of these individuals, is to reduce weight and increase baseline physical activity.

**Disclosure:**

No significant relationships.

